# Deleterious Mutations in the Mitogenomes of Cetacean Populations

**DOI:** 10.3390/biology15020199

**Published:** 2026-01-21

**Authors:** Matthew Freeman, Umayal Ramasamy, Sankar Subramanian

**Affiliations:** 1 Centre for Bioinnovation, School of Science, Technology, and Engineering, The University of the Sunshine Coast, Moreton Bay, QLD 4502, Australia; matthew.freeman@research.usc.edu.au; 2 Australian Research Centre for Human Evolution, Griffith University, Nathan, QLD 4111, Australia; u.ramasamy@griffith.edu.au

**Keywords:** deleterious mutations, effective population size, genetic drift, IUCN Red List, Cetacea, dN/dS ratio

## Abstract

Cetaceans are artiodactyls adapted to live in the marine environment, and this group includes whales, dolphins, and porpoises. Although mitochondrial nucleotide diversity has been reported separately for many cetacean groups, the proportion of deleterious mutations in these populations is unknown. Furthermore, a comparison of mitogenomic diversities across all cetaceans is also lacking. To investigate this, we conducted a comparative genomic analysis of 2244 mitochondrial genomes from 65 populations across 32 cetacean species. We observed a 78-fold variation in mitogenomic diversity among cetacean populations, suggesting a large difference in genetic diversity. We used the ratio of nonsynonymous-to-synonymous diversities (dN/dS) to measure the proportion of deleterious mutations in the mitochondrial exomes. The dN/dS ratio showed a 22-fold difference between the cetacean population. Based on genetic theories, the large differences observed in the two measures could be attributed to differences in the effective sizes of the cetacean populations. Typically, small populations have low heterozygosity and a high dN/dS ratio, and the reverse is true for large populations. This was further confirmed by the negative correlation observed between heterozygosity and dN/dS ratios of cetacean populations. While our analysis revealed similarities in mitogenomic diversity between the endangered and least-concern cetacean species, the dN/dS ratio of the former was found to be higher than that of the latter. The findings of this study are useful for identifying the relative magnitude of reductions in the population sizes of different cetacean species. This will help conservation management efforts prioritise the use of limited resources, time, and effort to protect the cetacean populations that need immediate attention.

## Simple Summary

Here, we examined genetic diversity and the fraction of deleterious mutations by analysing 2244 mitochondrial genomes from 65 populations across 32 cetacean species, including whales, dolphins, and porpoises. The ratio of nonsynonymous-to-synonymous diversities (dN/dS) was used as the proxy for the proportion of deleterious mutations. Our results revealed a 78-fold variation in mitogenomic diversity and a 22-fold difference in the dN/dS ratio among the cetacean populations. The large differences observed in the two measures suggest a substantial variation in the effective sizes of cetacean populations. We also observed a negative relationship between genetic diversity and dN/dS ratios. These results suggest that small cetacean populations have low diversity and a low dN/dS ratio, whereas the reverse pattern holds for large populations. Our analysis revealed a high dN/dS ratio for endangered species and a low ratio for cetaceans of least concern. The observations of this study could be useful for the conservation management of marine mammals.

## 1. Introduction

The infraorder Cetacea refers to aquatic marine mammals, including whales, dolphins, and porpoises, that are distributed throughout the world’s oceans and freshwater bodies, inhabiting a diverse array of habitats [1]. Most of the cetaceans have been hunted by humans over a long period of time, and during the industrialisation era, the advent of steam ships and explosive harpoon guns enabled large-scale whaling in all major oceans [2]. This significantly reduced the effective population sizes (*N_e_*) of most cetaceans. It is well known that population size reduction causes population bottlenecks and an increased rate of inbreeding [3,4]. This results in reduced heterozygosity within cetacean populations. For example, the isolated population of Fin Whales in the Gulf of California (GOC) was further reduced by industrial whaling, resulting in a severe decline in heterozygosity [5].

Importantly, the reduction in *N_e_* leads to the accumulation of deleterious mutations in nuclear and mitochondrial genomes [4]. Many previous studies have quantified the mutation loads in terrestrial mammals using the ratio of diversities (dN/dS) at nonsynonymous (dN) and synonymous sites (dS) [6,7,8,9,10,11,12,13,14,15,16,17]. Nonsynonymous mutations can harm the organism as they change the amino acid encoded by the codon, and in contrast, synonymous mutations are neutral or harmless as they do not change the amino acid encoded. Therefore, the ratio of these two reflects the proportion of harmful nonsynonymous mutations present in a population. Using this measure, previous studies compared the mutation loads of large and small or bottlenecked populations. For instance, a study comparing eastern gorillas before and after a severe population decline observed an elevated proportion of deleterious nonsynonymous mutations in the present population owing to a bottleneck [16]. Similar elevated dN/dS ratios were observed for island dingoes [9], island foxes [12], and island kakapos [6] in comparison with their mainland cousins. Higher dN/dS ratios were also observed for landlocked salmons compared to their ocean counterparts [15]. Since the *N_e_* of island (or landlocked) populations are typically smaller than their mainland counterparts, the higher dN/dS ratios are caused by a reduction in *N_e_*. The process of domestication also introduces a severe bottleneck. This is evident from the elevated dN/dS ratio observed for the domesticated dog [11], silkworm [10], yak [18], rice [19] and sunflower [20] compared to their wild counterparts.

The mitochondrial genomes of most cetaceans have been sequenced, and this data was used to investigate their heterozygosity and phylogenetic relationship. However, the deleterious mutation load of cetacean mitogenomes has not been studied previously. Furthermore, most earlier studies were conducted on independent cetacean species, genus or families. No study has compared their diversity and the proportion of deleterious mutations across all cetaceans. Such a study is necessary as it will reveal the magnitude of difference in these measures across species and families. This is important for future conservation management strategies to estimate the extinction risk of each cetacean species and population. This is because it is well documented that accumulation of deleterious mutation load leads to eventual extinction, as observed for the Wrangel Island woolly mammoth [13].

Therefore, we conducted a comparative analysis of 2244 mitochondrial genomes from 32 cetacean species. We identified the populations within species and compared the diversity and the fraction of deleterious mutations across all cetacean populations. We also obtained the IUCN conservation status for these species and compared the proportions of deleterious mutations across species belonging to conservation statuses.

## 2. Methods

### 2.1. Genome Data

The sequences of assembled and annotated complete mitogenomes of all available Cetacean species were downloaded from the National Center for Bioinformatics Information [21]. This dataset consisted of 2347 completed mitochondrial genomes. Species with fewer than 5 individual genomes were excluded, reducing the dataset to 2253 genomes. The downloaded GenBank files were checked for 13 protein-coding DNA sequences (protein-CDS), and only genomes containing all 13 mitochondrial protein-CDS were included for further downstream analysis. This filtration removed 9 genomes, leaving a final dataset of 2244 mitogenomes from 32 species of Cetaceans and 8 families (Table 1).

### 2.2. CDS Extraction and Alignment

**Figure 1:**
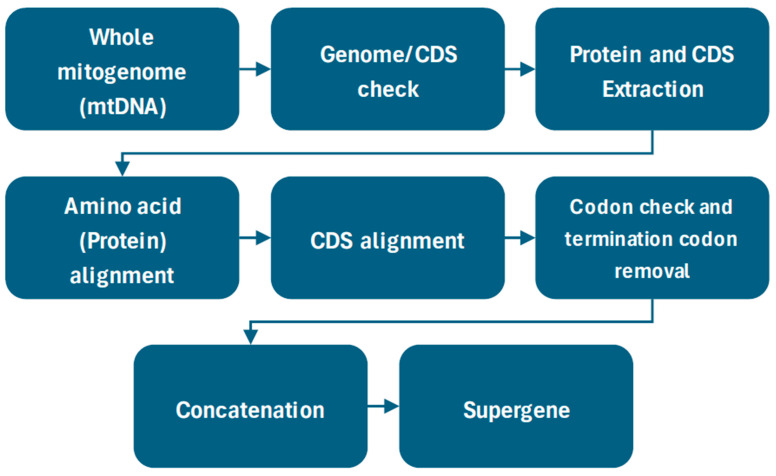
Workflow starting from genome download to the final CDS alignment.

The amino acid (protein) and CDS sequences were extracted from the protein-coding genes. We included only 12 genes coded in the heavy strand (ATP6, ATP8, COX1, COX2, COX3, CYTB, ND1, ND2, ND3, ND4, ND4L, and ND5). We excluded the ND6 gene on the light strand, as its base composition and mutation patterns are drastically different from those of the other 12 genes on the heavy strand [22,23]. The extracted protein sequences of the genes were aligned using the programme MUSCLE ver 3.8 [24], and the CDS alignment was created using the protein alignments as a guide. This method generated the CDS alignment while preserving the codons within their correct reading frames. The termination codons present at the end of the CDS were removed as they are not useful in estimating heterozygosity or dN/dS ratio, and codons containing even a single ‘N’ (unknown) nucleotide were also removed. The nucleotides of the 12 protein-coding genes were concatenated into a single supergene, which was then used for further downstream analyses (Figure 1).

### 2.3. Identification of Distinct Populations

The species in our Cetacean dataset consist of genomes of 2244 individuals, which may belong to one or more populations within each species. To identify distinct populations, we converted the supergene in *fasta* format to Variant Call Format (VCF) files containing only the variable sites of the genomes using a custom script. The Multidimensional scaling (MDS) method was then used to group the genomes based on their genetic relatedness using the software *Plink* ver. 1.9 [25]. The MDS files were used to produce a scatter plot for each species, and groupings were manually curated via visual inspection using a custom Graphical User Interface (GUI). This allowed manual editing of the groups, removing the genomes located between two groups, which could be the result of introgression. These analyses identified 52 distinct populations across 19 species, and the remaining 13 species had only a single population (which may also be due to limited sampling) (Table 1).

### 2.4. Genome Data Analysis

The supergene alignment was used to calculate nucleotide diversity within a population, which was estimated using the Nei and Li method [26]. The diversities at the nonsynonymous (dN) and synonymous positions (dS) were calculated using the Pamilo-Bianchi-Li method [27,28]. All these calculations were performed using the command-line version of the software MEGA-CC ver. 11 [29], and positions in alignments with gaps were excluded using the complete-site deletion option. To compute the standard error, the bootstrap method with 1000 pseudo-replicates was used.

### 2.5. Conservation Status

The conservation status for each species was obtained from the IUCN Red List (https://www.iucnredlist.org/ accessed on 10 September 2025), except for the Burrunan dolphin (*Tursiops australis*), which lacks an IUCN assessment, the conservation status was sourced from the Victoria state government [30] (accessed on 15 October 2025). We used only the global conservation assessments for 51 species, except for the Burrunan dolphin, for which a local assessment was utilised. The conservation status for each species followed the IUCN Red List categories: NE, Not Evaluated; DD, Data Deficient; LC, Least Concern; NT, Near Threatened; VU, Vulnerable; EN, Endangered; CR, Critically Endangered; EW, Extinct in the Wild, and EX, Extinct. Species that were Data-deficient or not evaluated were excluded from analyses. The remaining species were grouped into three categories: Least Concern (LC), Vulnerable and Near Threatened (VU + NT), and Endangered and Critically Endangered (EN + CR). These three groups were named as low, medium, and high-risk (for extinction). Note that there was no extinct cetacean species in our dataset.

## 3. Results

### 3.1. Mitogenomic Diversity

**Figure 2:**
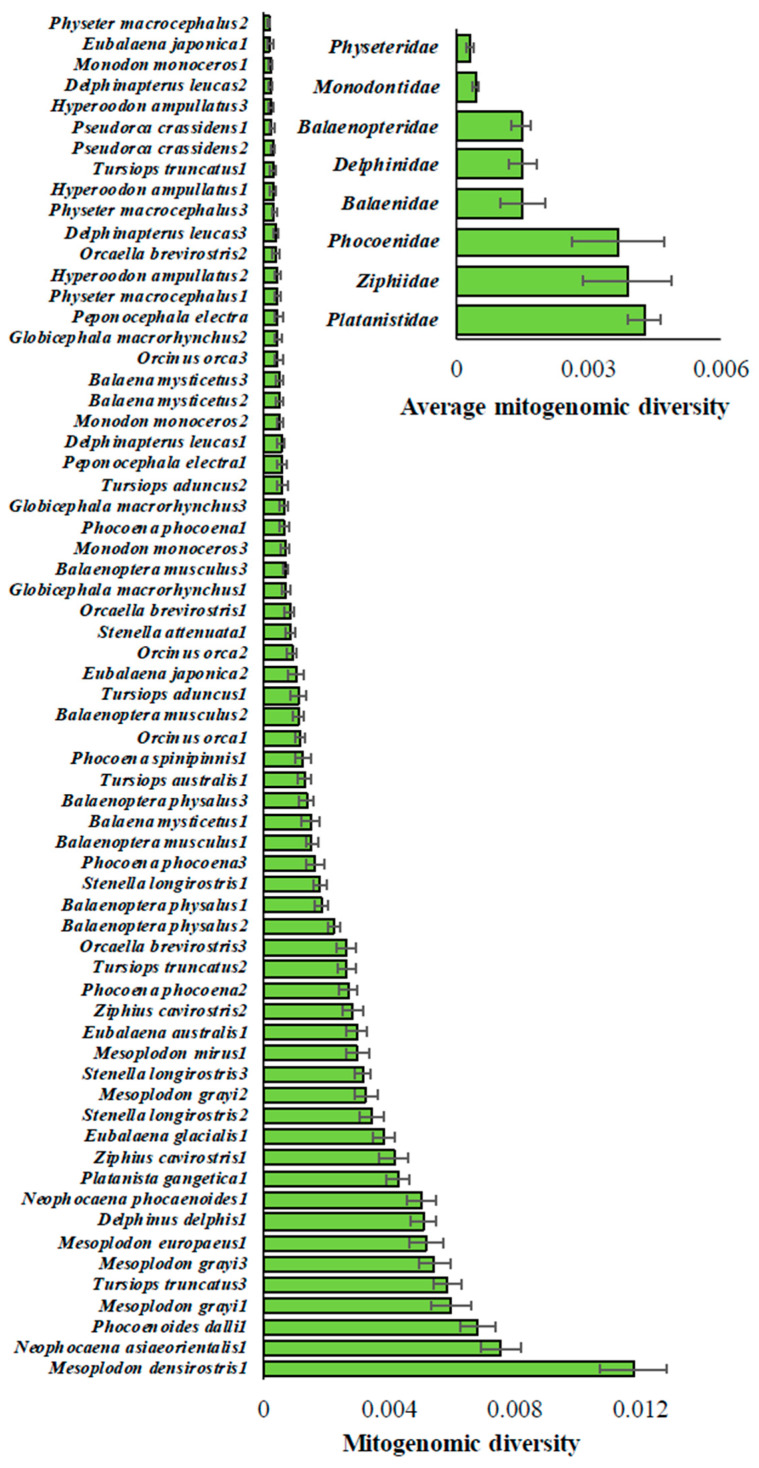
Mitogenomic diversity estimated for 65 cetacean populations. The error bars show the standard error of the mean. Inset: The average mitogenomic diversity estimated for each cetacean family. The number of genomes per family is given in Table 1.

Nucleotide heterozygosity was estimated using the concatenated supergene containing 12 mitochondrial protein-coding genes for each Cetacean species. Overall, the mitogenomic diversity showed a 78-fold difference among the species (Figure 2). While the highest diversity was observed for one of the Blainville’s beaked whale populations (*Mesoplodon densirostris*), as 0.0118, the lowest was recorded for one of the Sperm whale populations (*Physeter macrocephalus*) as 0.00015. After excluding the outlier (*Mesoplodon densirostris*), this difference was 50-fold. The average nucleotide diversity for the species within families also revealed a twelve-fold difference (Figure 2—inset). Sperm whales (*Physeteridae*) had the smallest (0.00058) and beaked whales (*Ziphiidae*) had the largest mean mitogenomic diversity (0.0072).

**Figure 3:**
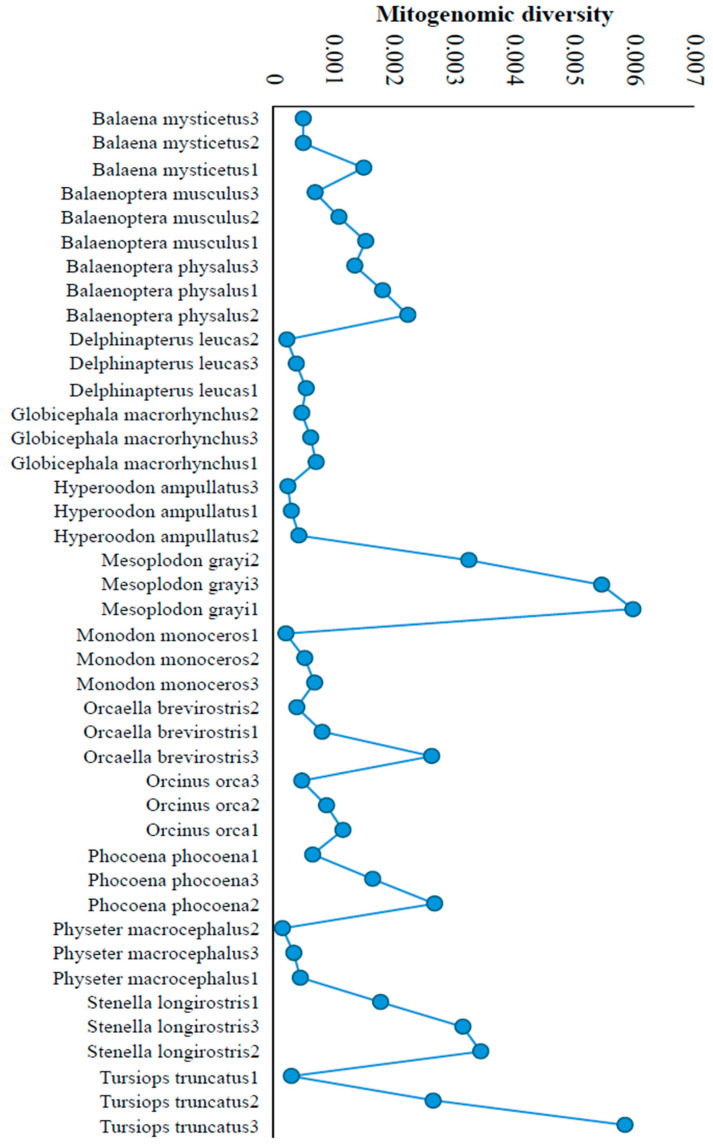
Mitogenomic diversity was computed for cetacean species with three populations. This shows the similarities or differences in diversity among populations of the same species. The populations within each species were placed in the ascending order of their diversity.

For most Cetacean species, diversity was largely similar across their populations (Figure 3). For example, these values for the three Beluga whale (*Delphinapterus leucas*) populations range between 0.0002 and 0.0005, and for the northern bottlenose whale (*Hyperoodon ampullatus*) populations, they range from 0.0002 to 0.0004. On the contrary, some populations, such as the common bottlenose dolphin (*Tursiops truncatus*), showed a 19-fold difference in diversity (0.0003–0.0058). Similarly, a 7-fold difference in diversity was observed among Irrawaddy dolphin (*Orcaella brevirostris*) populations (0.00039–0.00263).

### 3.2. Deleterious Mutations in Cetacean Populations

**Figure 4:**
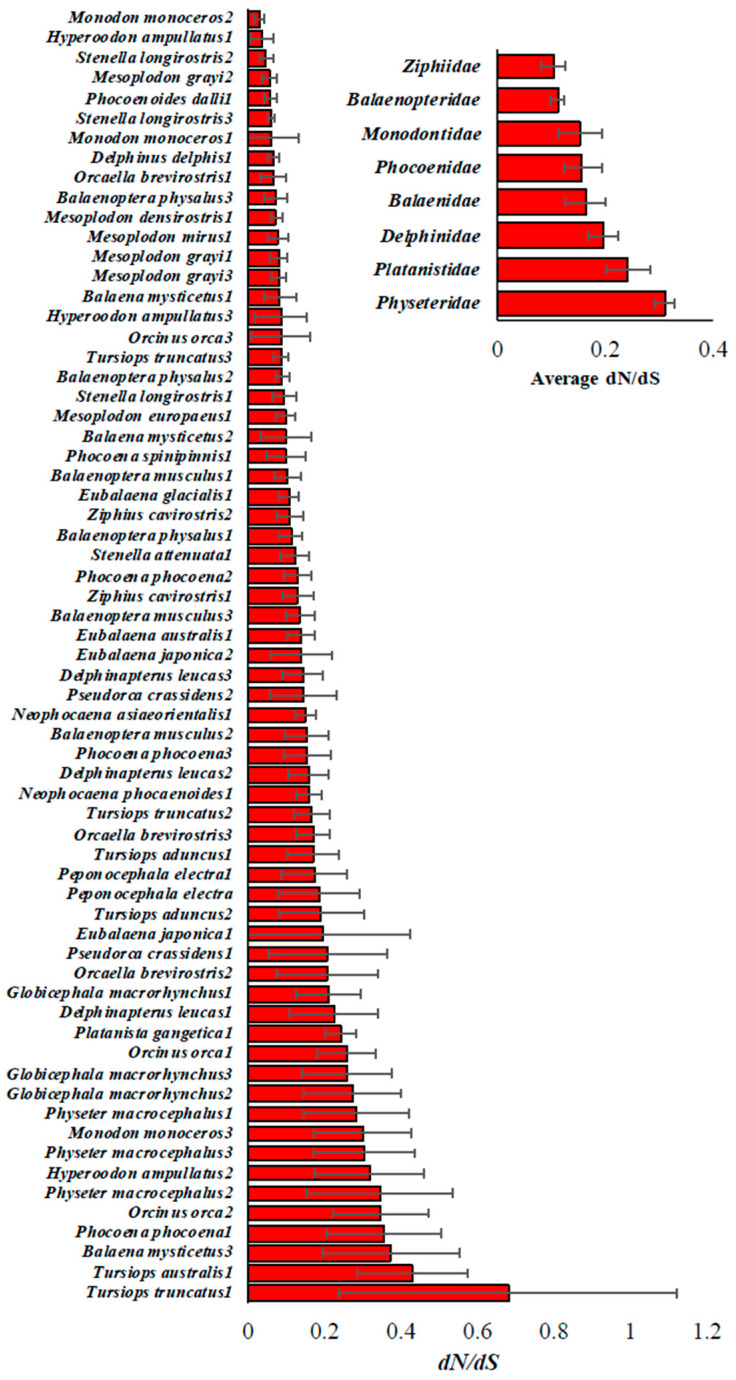
The dN/dS ratios were estimated for 65 cetacean populations. The error bars show the standard error of the mean. Inset: The average dN/dS ratio estimated for each cetacean family. The number of genomes per family is given in Table 1.

Synonymous mutations are neutral or harmless, whereas nonsynonymous mutations may be deleterious. Therefore, the ratio of diversities at nonsynonymous and synonymous sites (dN/dS) measures the proportion of deleterious nonsynonymous mutations segregating in a population. The dN/dS ratios estimated for the Cetacean populations showed a 22.6-fold difference (Figure 4). However, after removing the outlier (0.68—*Tursiops truncatus*), this difference was 14.3-fold. After the exclusion of the outlier, the dN/dS ratio of Burrunan dolphin (*Tursiops australis*) had the highest value (0.43), whereas Spinner dolphin (*Monodon monoceros*) had the lowest ratio (0.03). The mean dN/dS ratio estimated for Cetacean families showed a 4-fold difference. While Sperm whales (*Physeteridae*) accumulated the highest proportion of deleterious mutations (dN/dS = 0.33), and the *Balaenopteridae,* known also as Rorquals, had the smallest fraction of these mutations (dN/dS = 0.082) (Figure 4—inset).

**Figure 5:**
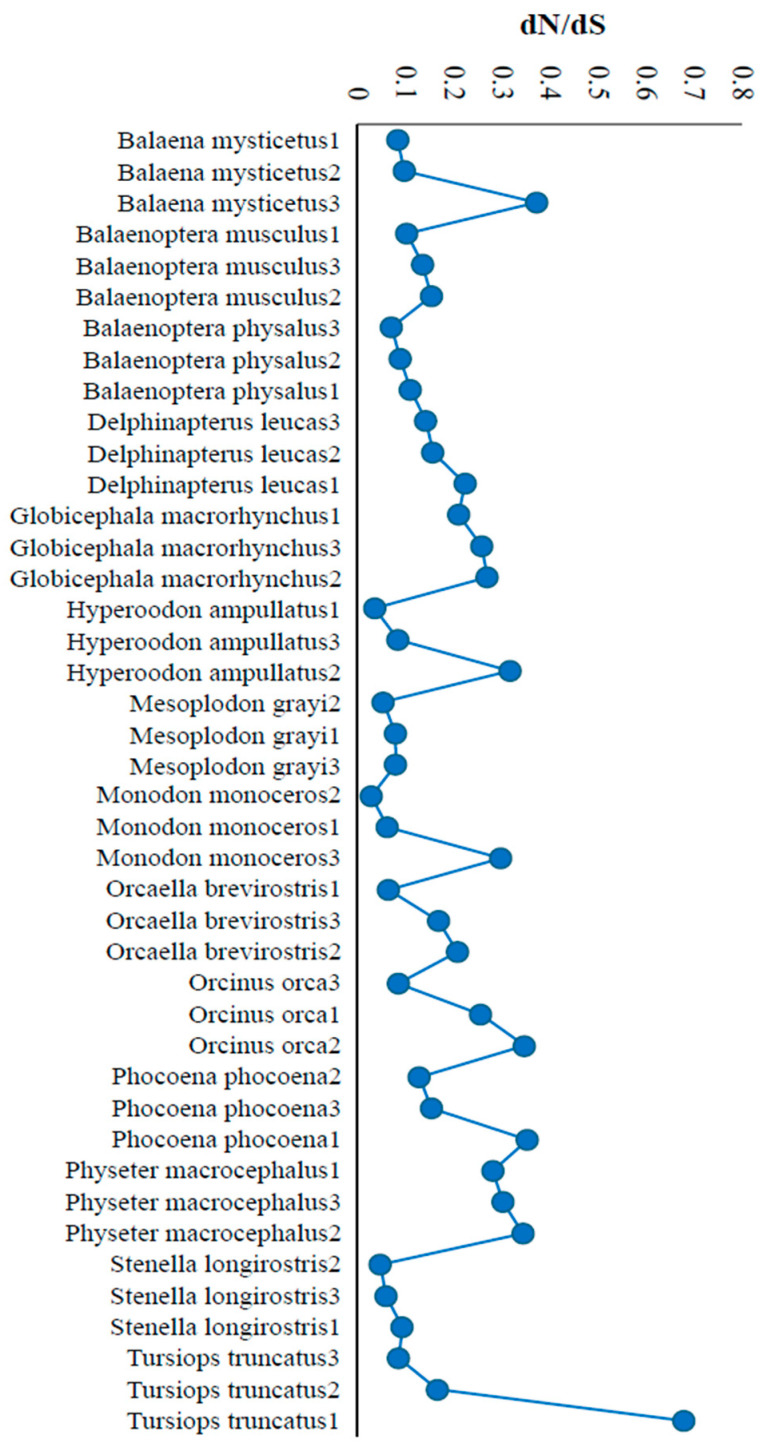
The ratios of dN/dS were calculated for the cetacean species with three populations. This shows the similarities or differences in dN/dS among the populations of the same species. The populations within each species were placed in the ascending order of the dN/dS ratios.

Most of the dN/dS ratios were also similar among the populations of the same species (Figure 5). There were a few exceptions. The narwhal (*Monodon monoceros*) showed a 10-fold difference in the dN/dS ratio across populations (0.029 to 0.299). This ratio varied 8.5 times between the populations of northern bottlenose whale (*Hyperoodon ampullatus*) (0.037 to 0.32). Similarly, the common bottlenose dolphin (*Tursiops truncatus*) populations showed a 7.8-fold difference in dN/dS estimates (0.087 to 0.68).

### 3.3. Relationship Between Mitogenomic Diversity and the Fraction of Deleterious Mutations

**Figure 6:**
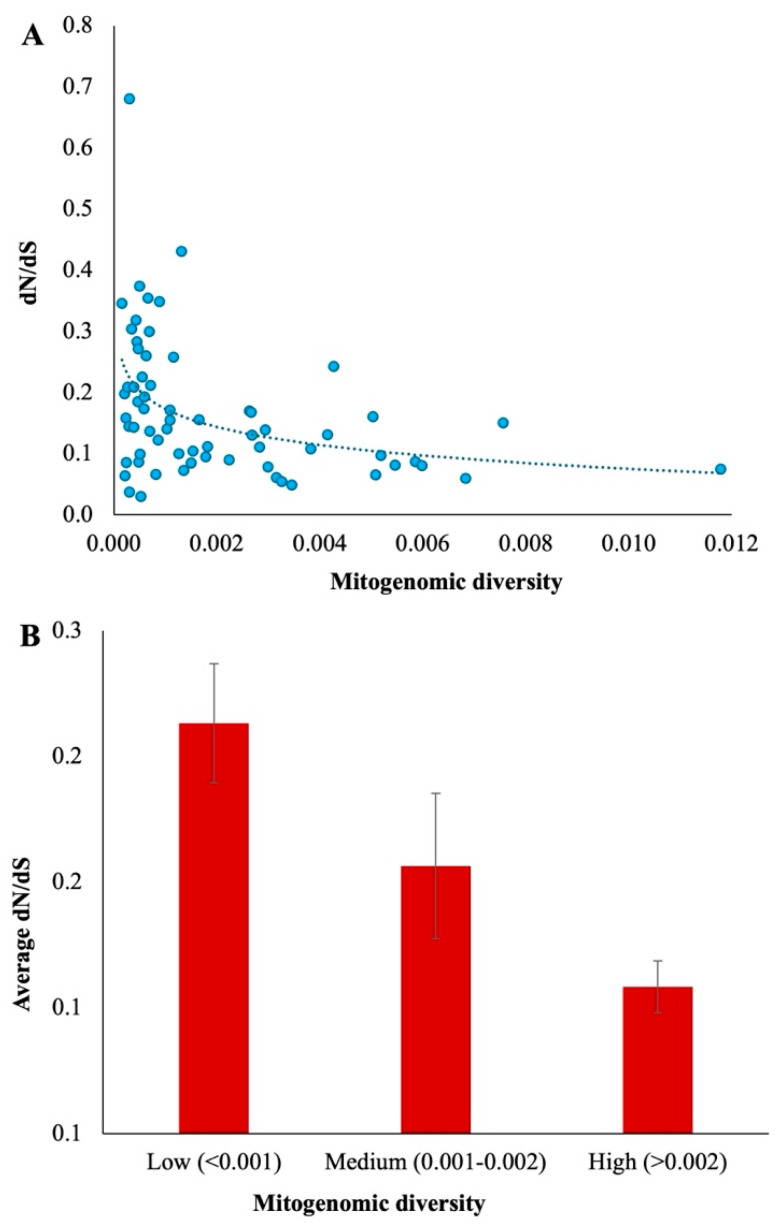
(**A**) Correlation between mitogenomic diversities and mutation load (dN/dS ratios) estimated for each cetacean population. The relationship is highly significant (*r* = −0.43, *p* = 0.0004). The best-fitting curve is shown. (**B**) The average dN/dS ratio was estimated for cetacean populations with low (<0.001), medium (0.001–0.002), and high (>0.002) levels of mitogenomic diversity. The error bars show the standard error of the mean.

To investigate the relationship between mitogenomic diversity and dN/dS ratio, we plotted these estimates against each other. This analysis produced a significant negative relationship between the two variables (Figure 6). A log curve best fitted this relationship. The correlation was highly significant with a Pearson correlation coefficient (*r*) of −0.41 and *p* = 0.0007. Since the distribution of this date is not well known, we also examined the Spearman rank correlation. This analysis also produced a highly significant negative relationship (*r* = −0.43, *p* = 0.0004). This suggests that populations with low diversity have a high dN/dS ratio, whereas those with high diversity have a low dN/dS ratio. To further investigate this, we also divided the dataset into three groups based on the level of mitogenomic diversity: Low (<0.001), Medium (0.001–0.002), and High (>0.002), and estimated the average dN/dS ratio for the populations belonging to these categories. The results showed that on average, populations with low diversity have a two-fold higher dN/dS ratio than those with high heterozygosity (0.213 vs. 0.108).

### 3.4. Mutation Load and IUCN Red List Status

**Figure 7:**
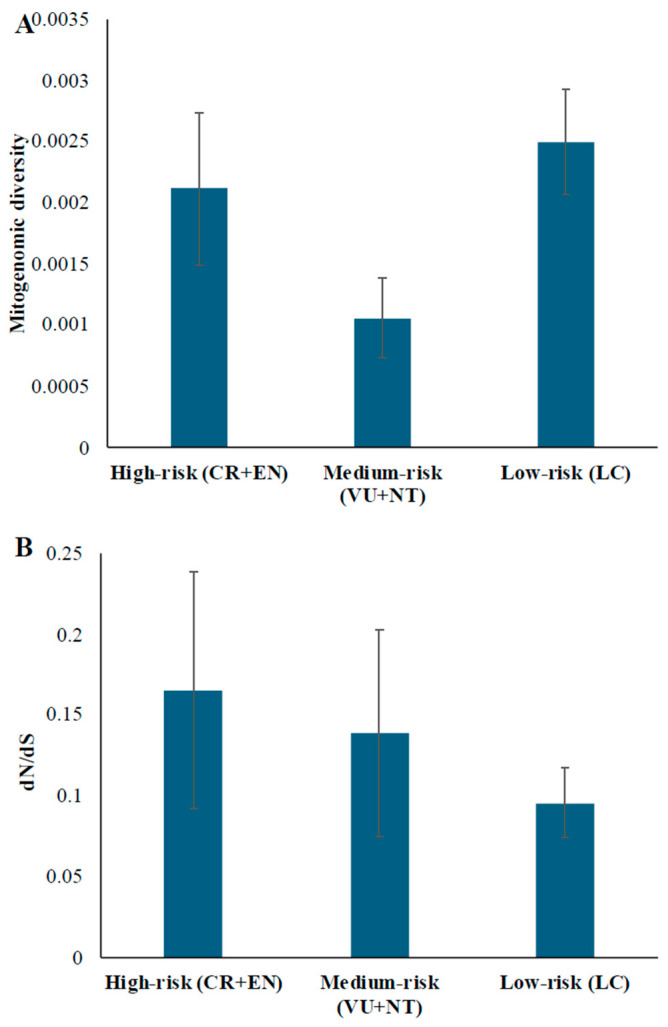
The average mitogenomic diversity (**A**) and dN/dS ratio (**B**) estimated were for cetacean populations with low, medium, and high extinction risk. The IUCN Red List statuses were used to define these groups. The high-risk group includes Critically Endangered (CR) and Endangered (EN) species. While the medium-risk group includes Vulnerable (VU) and Near-Threatened (NT) species, the low-risk group includes Least Concern (LC) species. The error bars show the standard error of the mean.

Finally, to investigate the relationship between deleterious mutational load and conservation status of species, we obtained the IUCN Red List status for each species. These are ordered from lowest to highest level threat of extinction: LC (Least Concerned), NT (Near threatened), VU (Vulnerable), EN (Endangered), and CR (Critically Endangered). Due to the limited number of data, we combined the populations belonging to CR + EN and VU + NT and named the resulting groups as high-risk and medium-risk (for extinction). We then compared the mean diversity (Figure 7A) and dN/dS ratio (Figure 7B) of these groups with the average estimates obtained for the LC (low-risk) group. Contrary to the expectation, the heterozygosity of the high-risk group (0.0021) was higher than that of the medium-risk group (0.0011), and the heterozygosities of the high-risk and low-risk groups were similar (0.0021 and 0.0025, respectively). In contrast, the average dN/dS ratio of the high-risk group (0.165) was higher than that of the medium-risk group (0.139), which in turn is higher than that of the low-risk group (0.096).

## 4. Discussion

In this study, we compared the mitochondrial genetic diversity and the proportion of deleterious mutations across all Cetacean populations for which the data were available. We found a 78-fold difference in the nucleotide diversity between cetacean populations. It is well known that diversity, or heterozygosity, is determined by the product of mutation rate (μ) and effective population size (*Ne*). The mutation rate is expected to be similar across the species within a mammalian order. A recent study on Humpback whales (*Megaptera novaeangliae*) observed a mutation rate of 1.12 (0.94 − 1.3) × 10^−8^ [31]. This estimate was similar to the rate of 1.11 (0.97 − 1.25) × 10^−8^ estimated for three baleen whales (Fin whale—*Balaenoptera physalus*, blue whale—*Balaenoptera musculus,* and bowhead whale—*Balaena mysticetus*) [31]. Another study also estimated similar mutation rates for killer whales and bottlenose dolphins, which were 0.9 × 10^−8^ and 1.4 × 10^−8^, respectively [32]. Therefore, the observed vast difference in diversity is more likely be attributed to effective population size. Therefore, diversity directly informs us of the effective size of cetacean populations and could therefore be a good indicator for monitoring their conservation status.

Our results showed a 22-fold difference in the dN/dS ratios between cetacean populations. The fraction of deleterious mutations is expected to be higher in small populations than in large populations [33,34]. This is because purifying selection is less efficient in removing deleterious mutations in small populations due to the strong effects of genetic drift. Therefore, the most likely explanation for the large difference in dN/dS ratios is the variation in effective sizes between cetacean populations. Previously observed higher dN/dS ratios of island populations of dingoes [9], foxes [12], Orkney voles [35], and landlocked salmon were attributed to their small population sizes resulting from habitat limitation [15]. Furthermore, higher dN/dS ratios were observed in the declining mammoth population [13]. An earlier study showed a positive correlation between generation time and deleterious mutation load in mitochondrial genomes of mammals [36]. This study used generation time as the proxy for effective population size, and mammals with long generation times have small population sizes. Therefore, this study provided evidence for the negative correlation between effective population size and deleterious mutation load.

We also showed a negative correlation between diversity and dN/dS ratios estimated for the cetacean populations. This relationship suggests that small populations typically have high proportions of deleterious mutations and large populations have low proportions. Therefore, diversity and mutation loads are the two indicators of effective population size. This further confirms our suggestion that the mutation rate is largely similar across cetaceans, and the population size variation predominantly explains the patterns observed in this study. Similar correlations between genomic diversity and dN/dS ratios were reported in dingoes [9], dogs [11], cows [37], and island foxes [12].

The long-term reduction in the population size is predicted to increase the risk of extinction of a population or species [38]. This is because a reduction in the population size leads to the accumulation of deleterious mutations. Furthermore, population size reduction increases inbreeding, which further elevates the deleterious mutation load. This effect is more pronounced in mitochondrial genomes, which do not recombine, leading to mutational meltdown [3,39]. The relationship between genetic diversity and extinction risk has been debated. While some studies provide support for this [38,40], others do not [41,42]. The results of this study did not support the expected association between heterozygosity and extinction risk. Typically, endangered species are expected to have lower heterozygosity than the vulnerable and least concerned categories. However, we found that the mitogenomic diversity of endangered cetaceans was higher than that of vulnerable and near-threatened groups and was similar to that of least concerned cetaceans. In contrast, we found that the fraction of deleterious mutations is higher in endangered (high-risk) and vulnerable (medium-risk) species of Cetacea than in species belonging to the least concerned (low-risk) category. Although we see a clear trend between mutation loads and IUCN Red List status, the differences were not highly significant due to the small sample sizes, particularly for the high-risk species. Hence, further research using a large dataset is needed.

Our study has important implications for conservation management. Out of 31 cetacean species for which the IUCN Red List status was available, 45% (14) were under different levels of extinction risk (CR, EN, VU, or NT). Importantly, 23% (7) were either endangered or critically endangered. Since we showed a large variation in the two predictors of effective population sizes (diversity and the fraction of deleterious mutations), this is useful for assessing the historical demographic status of various cetaceans. This will help identify the cetacean populations and species that are declining. Therefore, this information will help conservation management agencies prioritise strategic practices and allocate resources to the species/populations that need immediate attention.

## 5. Conclusions

In this study, we investigated the mitochondrial genetic diversity and the fraction of deleterious mutations (dN/dS ratio) in cetacean populations. Our results showed a substantial variation in diversity and dN/dS ratio across 65 populations belonging to 32 cetacean species. The observed correlation between these two measures suggests that small cetacean populations have low diversity and a high fraction of deleterious mutations, whereas large ones have high diversity and a low proportion of harmful mutations. The high dN/dS ratio observed in endangered cetacean species indicates the accumulation of harmful mutations in their mitochondrial genomes. These results are helpful in advising conservation management agencies.

## Figures and Tables

**Table 1:** Cetacean species, number of genomes and populations used in this study.

Common Name (Scientific Name)	Genomes	Populations
**Balaenidae**		
Bowhead whale (*Balaena mysticetus*)	50	3
North Atlantic right whale (*Eubalaena glacialis*)	14	1
North Pacific right whale (*Eubalaena japonica*)	29	2
Southern right whale (*Eubalaena australis*)	17	1
**Balaenopteridae**		
Blue whale (*Balaenoptera musculus*)	182	3
Fin whale (*Balaenoptera physalus*)	155	3
**Delphinidae**		
Burrunan dolphin (*Tursiops australis*)	8	1
Common bottlenose dolphin (*Tursiops truncatus*)	136	3
Common dolphin (*Delphinus delphis*)	34	1
False killer whale (*Pseudorca crassidens*)	60	2
Indo-Pacific bottlenose dolphin (*Tursiops aduncus*)	24	2
Irrawaddy dolphin (*Orcaella brevirostris*)	88	3
Killer whale (*Orcinus orca*)	153	3
Melon-headed whale (*Peponocephala electra*)	33	2
Short-finned pilot whale (*Globicephala macrorhynchus*)	100	3
Spinner dolphin (*Stenella longirostris*)	104	3
Pantropical spotted dolphin (*Stenella attenuata*)	70	1
**Monodontidae**		
Beluga whale (*Delphinapterus leucas*)	545	3
Narwhal (*Monodon monoceros*)	70	3
**Phocoenidae**		
Burmeister’s porpoise (*Phocoena spinipinnis*)	7	1
Dall’s porpoise (*Phocoena dalli*)	7	1
Finless porpoise (*Neophocaena phocaenoides*)	9	1
Harbour porpoise (*Phocoena phocoena*)	51	3
Yangtze finless porpoise (*Neophocaena asiaeorientalis*)	9	1
**Physeteridae**		
Sperm whale (*Physeter macrocephalus*)	67	3
**Platanistidae**		
Ganges river dolphin (*Platanista gangetica*)	10	1
**Ziphiidae**		
Blainville’s beaked whale (*Mesoplodon densirostris*)	12	1
Cuvier’s beaked whale (*Ziphius cavirostris*)	22	2
Gervais’s beaked whale (*Mesoplodon europaeus*)	8	1
Gray’s beaked whale (*Mesoplodon grayi*)	23	3
True’s beaked whale (*Mesoplodon mirus*)	10	1
Northern bottlenose whale (*Hyperoodon ampullatus*)	137	3
Total	2244	65

## Data Availability

The original data presented in the study are openly available at https://www.ncbi.nlm.nih.gov/genbank/ (accessed on 5 June 2025). The in-house Python (ver. 3.12) scripts used in this study are available upon request.
